# Greening the Production of Indigo Blue Exploiting Light and a Recombinant *Synechocystis* sp. PCC6803 Strain Expressing the Enzyme mFMO

**DOI:** 10.1111/1751-7915.70146

**Published:** 2025-04-28

**Authors:** Giovanni Loprete, David Rubert, Francesco Bellusci, Nikola Lončar, Marco W. Fraaije, Elisabetta Bergantino

**Affiliations:** ^1^ Synthetic Biology and Biotechnology Unit, Department of Biology University of Padova Padova Italy; ^2^ Gecco Biotech Groningen the Netherlands; ^3^ Molecular Enzymology Group University of Groningen Groningen the Netherlands

**Keywords:** biocatalysis, biotransformation, flavin‐containing monooxygenase, indigo, *Synechocystis* sp. PCC6803

## Abstract

Cyanobacteria are emerging as interesting cell factories, offering the significant advantage of their in‐built photosynthetic machinery, which generates NADPH to support redox biocatalysis. In this study, we assessed the potential of the cyanobacterium *Synechocystis* sp. PCC6803 in producing the dye indigo by light‐driven whole‐cell biotransformation using indole as a starting compound. A stable transgenic strain expressing a flavin‐containing monooxygenase from *Methylophaga aminisulfidivorans* (mFMO) was engineered, enabling light‐dependent indigo production. Upon optimising conditions, effective biotransformations could be performed, resulting in 112 mg/L indigo (86% conversion of the furnished indole). Additionally, we present a method for the recovery of the secreted dye directly from the growth medium through solid‐phase absorption on polyamide nets. Overall, the effectiveness and sustainability of the biotransformation in *Synechocystis* sp. PCC6803 performed at the laboratory scale provide a strong basis for further exploring the applicability of the process.

## Graphical abstract

We developed a sustainable lab‐scale method for producing bio‐indigo using an engineered *Synechocystis* sp. PCC6803 strain expressing the enzyme mFMO and light. Optimised growth conditions yielded 112 mg/L of bio‐indigo. A new purification technique using polyamide nets allows efficient recovery from the culture medium, reducing environmental impact.

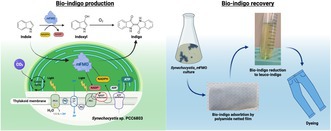

## 1 Introduction

Indigo is the oldest and most widely consumed vat dye in the textile industry, and it is also employed in traditional Chinese medicine for several gastrointestinal disorders, skin diseases and asthma (Sun et al. 2021). Natural indigo has been used since ancient times as extracted from different plants such as *Indigofera tinctoria* , *Persicaria tinctoria* and *Isatis tinctoria* . With the exponentially increasing demand for dyes, the natural extraction process gradually declined from 19th century and has been replaced by chemical synthesis (Mocquard et al. 2022). Currently, around 80,000 tons of indigo are chemically produced each year with the use of non‐renewable petrochemicals and toxic compounds, and the generation of dangerous pollutants. The predominant synthetic process in industry starts from aniline and continues with the indole ring closure, and proceeds through a series of reactions based on hazardous chemicals such as HCHO, HCN and NaNH_2_. Despite the simplicity of this process and its high productivity and effectiveness in terms of both purity and cost, the chemical synthesis of indigo results in toxic by‐products from the use of strong reducing agents and metal catalysts (Blackburn et al. 2009). Furthermore, some synthetic steps are carried out under harsh conditions and high temperatures.

Nowadays, the increasing demand for industrial eco‐friendly processes is rising, and thus the production of so‐called bio‐indigo by biotechnological processes is receiving new attention. Enzyme‐based processes are characterised by some major advantages, such as mild reaction conditions and the use of renewable sources without the employment of toxic chemicals. Various enzymes, mostly belonging to the oxidoreductases family (EC 1), have been explored for indigo synthesis. These include naphthalene dioxygenases (NDO; EC 1.14.13.8), phenol hydroxylases (PHs; EC 1.14.13.7), cytochrome P450 monooxygenases (CYPs; EC 1.14.14.X), unspecific peroxygenases (UPOs; EC 1.11.2.1) and flavin‐containing monooxygenases (FMOs; EC 1.14.13.8) (Ensley et al. 1983; Banoglu et al. 2001; McClay et al. 2005; Han et al. 2011; Kalum et al. 2014).

Several strategies, including the employment of engineered heterotrophic microbes, have been proposed to produce indigo by fermentation or biotransformation, the latter mostly employing indole or L‐tryptophan as precursors (for a review see Chandel et al. 2024). Among the aforementioned enzyme classes, NADPH‐dependent FMOs have attracted particular interest due to the high reaction yields and the purity of the formed indigo (Ma et al. 2018; Fabara and Fraaije 2020; Fan et al. 2024). To date, the highest yields reported remain those of Berry et al. (2002), who obtained 18 g/L indigo by fermentation from glucose and 23 g/L by biotransformation of tryptophan using different metabolically engineered *Escherichia coli* strains expressing an NDO. Although many approaches were demonstrated to be valuable at the small scale, their translation to cost‐effective large‐scale bioprocesses is still far from established. A recent study by Linke et al. (2023) highlights the reasons behind this gap in data and thoroughly evaluates the practical aspects for the possible implementation of a platform for the production of bio‐indigo, validating its industrial potential.

Our study assesses for the first time the potential of the cyanobacterium *Synechocystis* sp. PCC6803 (hereafter *Synechocystis*) for indigo production under autotrophy. Cyanobacteria are unicellular, photosynthetic microalgae, studied for decades as model organisms for photosynthesis and that have developed as alternative and useful cell factories. Compared to the workhorse *E. coli* , they have the considerable advantage of requiring only light, water, some salts and CO_2_ as carbon sources for growth, representing an attractive host for developing eco‐friendly industrial processes. *Synechocystis* has gained great attention in recent years as a whole‐cell biocatalyst for the light‐driven oxidation of a broad range of substrates (Malihan‐Yap et al. 2022 and references therein). Notably, *Synechocystis'* genome is relatively easy to manipulate and can sustain the activity of NADPH‐dependent biocatalysts. NADPH is the most abundant reducing cofactor produced by cyanobacteria, being continuously regenerated through photosynthesis (Jodlbauer et al. 2021; Toepel et al. 2023). Despite these advantages, the employment of *Synechocystis* in biotransformations remains still limited to the laboratory scale.

**FIGURE 1:**
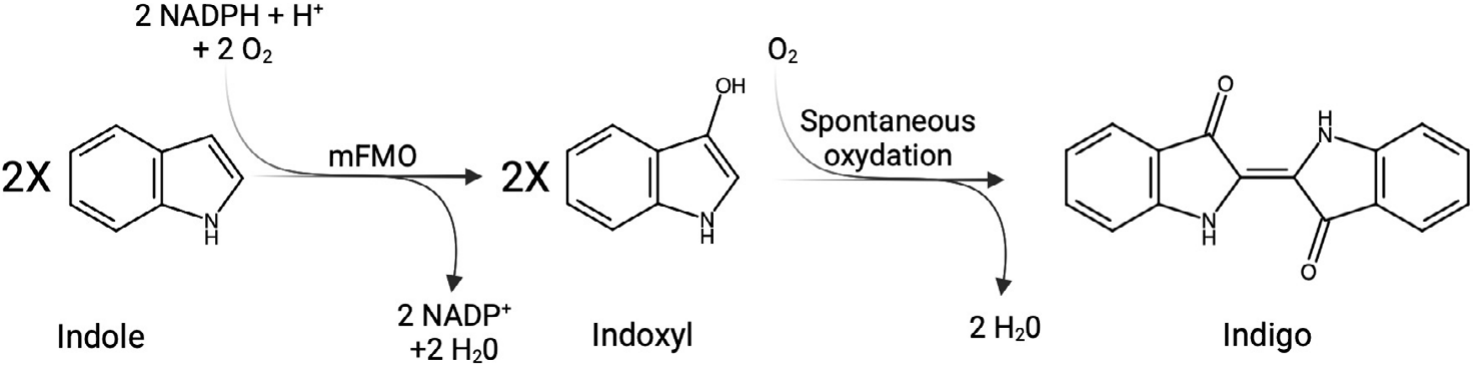
Schematic representation of the cascade reactions that result in bio‐indigo production by a recombinant *Synechocystis* expressing mFMO.

In this work, we describe the production of indigo by using a light‐fueled whole‐cell biotransformation carried out by a recombinant *Synechocystis* strain. We engineered a strain stably expressing the FMO from *Methylophaga aminisulfidivorans* (mFMO), which can oxidise indole into indigo using NADPH and dioxygen (Figure 1). We demonstrated that, in optimised conditions, *Synechocystis* tolerates 1.0 mM of the substrate indole, achieving a conversion to secreted indigo of 86%. Additionally, we also developed an in situ recovery method for the dye which enabled the recovery of 71% of the total produced indigo directly from the growth medium by capturing it on polyamide nets.

## 2 Experimental Procedures

### 2.1 Enzymes and Reagents

Polymerases, restriction enzymes, DMSO, substrate indole and the standard product indigo were purchased at Thermo Fisher Scientific (California, United States). DNA oligonucleotides (Table S1) and all other reagents were purchased at Sigma‐Aldrich (Steinheim, Germany). Styrene‐divinylbenzene beads (Bio‐Beads SM‐2) were purchased at Bio‐Rad (California, United States). Polyamide nets (PA‐38GG) were purchased at SEFAR AG (Heiden, Switzerland).

### 2.2 Bacterial Strains, Cloning and *Synechocystis* Transformation

The following strains were used: *Synechocystis* sp. PCC 6803 purchased at Pasteur Culture collection of Cyanobacteria (PCC) (Paris, France), and *E. coli* DH‐5α at New England Biolabs NEB (Massachusetts, United States).

Cloning in *E. coli* and PCR amplifications were performed by routine methodologies. A complete list of the oligonucleotides used is presented in Table S1.

Plasmid pSuperP_UV (Figure S1) was constructed by amplifying from the *Synechocystis* genome the DNA sequence spanning nucleotides 156,073–157,789 (primers NR1 and NR2); the amplimer was used as a template for nested PCRs inserting a PvuII site at the 5' end (primer PvuII_NS1_For), an internal PstI site (primers NS1_mut_Rev and NS2_mut_For) and contiguous DraI and PvuII sites at the 3' end (DraI_PvuII_NS2_Rev).

The resulting fragment (nucleotides 156,282–157,496 from the cyanobacterial genome) was cut by PvuII and cloned between the two PvuII sites present in the commercial vector pBS SK(+) (Agilent, United States) giving the plasmid called pBSSK(+)_NS:ndhB. Similarly, by subsequent PCRs, in parallel, the promoter P_cpc560_ (amplified by the primers SuperP_BglII_for and SuperP_NcoI_rev, introducing BglII and NcoI sites) and the terminator T_rbc_ (primers HistagRbcTer_for and RbcTer_rev, respectively introducing a XhoI and PstI+XhoI sites) were cloned respectively upstream and downstream of the MCS of the pET28a(+) vector (Merck KGaA, Darmstadt, Germany), exploiting unique sites in the vector and introduced sites in the amplimers. The obtained plasmid was called pET28a(+)_P_cpc560__T_rbc_. The fragment of its sequence defined by the BglII (5′ side) and PstI (3′ side) unique sites was transplanted between the BglII site (naturally occurring in the locus ssl0410 of the *Synechocystis* genome) and the engineered PstI site of plasmid pBSSK(+)_NS:ndhB, giving a third plasmid named pSuperp560_UniversalVector_NoRes. By cloning into its unique PstI site the kanamycin cassette taken from the commercial vector pUC4K (Amersham Bioscience, United Kingdom), plasmid pSuperP_UV was finally obtained and fully sequenced. It contains 7 DraI sites (Figure S1), two of them being at each side of the sequences for homologous recombination; the other 5 DraI sites are spread all along the backbone vector, thus destroying it after the restriction reaction.

The coding sequence for mFMO was obtained by PCR amplification from plasmid pCRE2‐mFMO (Rioz‐Martínez et al. 2011; Lončar et al. 2019) using primers FMO_for_NcoI and FMO_rev_NotI, introducing the NcoI and NotI sites at the 5′ and 3′ ends of the ORF, respectively. The two restriction sites were exploited for cloning the FMO coding sequence into pSuperP_UV to give the plasmid pSuperP_FMO.

The protocol for *Synechocystis* transformation, using plasmid DNAs digested by DraI, was the one based on phosphate deprivation described by Pope et al. (2020).

### 2.3 Standard Cultivation Conditions for *Synechocystis*

Standard BG11 medium used for *Synechocystis* cultivation was composed of: 10 mM N‐[Tris(hydroxymethyl)methyl]‐2‐aminoethanesulfonic acid (TES) pH 8.0, 6 mg/L ferric ammonium citrate, 30.5 mg/L K_2_HPO_4_, 20 mg/L Na_2_CO_3_, 2.86 mg/L H_3_BO_3_, 1.81 mg/L MnCl_2_, 0.22 mg/L ZnSO_4_, 0.39 mg/L NaMoO_4_, 0.08 mg/L CuSO_4_, 0.05 mg/L Co(NO_3_)_2_, 1.49 g/L NaNO_3_, 75 mg/L MgSO_4_, 36 mg/L CaCl_2_, 9.2 mg/L citric acid and 2.8 μM EDTA pH 8.0. Flasks and Corex tubes were covered with a hydrophobic cotton cap allowing air exchange while limiting medium evaporation. Standard culture cultivation was performed in continuous shaking (150 rpm) and under constant light illumination, 50 μmol photons •m^−2^ •s^−1^.

### 2.4 Total Protein Extraction From *Synechocystis* Cells and Western Blot Analysis

Expression of the mFMO endowed with a C‐terminal His‐tag in *Syn_*FMO was verified by Western blotting. Total protein extract was obtained by harvesting 10 mL of cell culture in exponential growth phase by centrifugation (8 min, 6000 g, at 4°C). The pellet was washed by resuspension in 1 mL of Washing Buffer (50 mM HEPES‐NaOH pH 7.5, 30 mM CaCl_2_) and recollected. Cells were resuspended in 1 mL of Resuspension Buffer (50 mM HEPES‐NaOH pH 7.5, 30 mM CaCl_2_, 800 mM sorbitol, 1 mM ε‐amino‐n‐caproic acid), then homogenised using One Shot Cell disruptors (Costant Systems, Daventry, United Kingdom).

Homogenate was fractionated by centrifugation (2 min, 1500 g, at 4°C).

Soluble protein content was quantified by Bradford assay (SERVA Electrophoresis GmbH, Heidelberg, Germany) and 10 μg were run in 12% UREA‐PAGE. Western Blot was performed using primary Mouse monoclonal anti‐His‐Tag antibody HRP‐conjugated (SB194b, Southern Blotting, Birmingham, USA). VWR Imager CHEMI Premium was used for revelation (VWR International s.r.l., Milan, Italy).

### 2.5 Monitoring Cyanobacterial Growth by Optical Density and Cell Counting

Cyanobacterial population density was estimated from the turbidity of the culture, typically expressed as OD_730_. Since *Synechocystis* naturally produces PHB under stress conditions (as in case of metabolic burden), we decided to test if the extra PHB produced by *Syn_*FMO could affect optical density measurements. Therefore, growth profiles were also evaluated by cell counting, comparing *Syn*_FMO with control strains. At fixed times, samples from cultures were diluted in the same ratio; 20 μL were deposited in the cell counting chamber, allowed to settle and counted using the Cellometer Auto X4 Cell Counter (Nexcelom Bioscience). Counts were performed three times on three independent replicates.

### 2.6 Transmission Electron Microscopy (TEM) Images

Samples for TEM analysis were prepared as follows. 2 mL of cell culture in the exponential growth phase were harvested by centrifugation (30 min, 1500 g at 4°C). Pellets were resuspended in 2.5% glutaraldehyde, 2% paraformaldehyde and 0.1 M sodium cacodylate. Samples were incubated for 48 h at 4°C in darkness. Then, samples were harvested by centrifuging for 30 min, 1500 g, and were resuspended in a 0.1 M sodium cacodylate solution. Post‐fixation was performed by 1% osmium tetroxide and 1% potassium ferrocyanide for 2 h at 4°C, ethanol dehydrated, then infiltrated in a mixture of EMbed 812 (Electron Microscopy Sciences) epoxy resin and propylene oxide 1:1 overnight, and finally embedded. Ultrathin sections were obtained with a Reichert‐Yung Ultracut ultramicrotome, collected on 200 mesh copper grids and subsequently counterstained with uranyl acetate and lead citrate. Samples were examined with a Tecnai G2 (FEI) transmission electron microscope operating at 120 kV, and digital images were acquired using a Veleta (Olympus Soft Imaging Solutions) digital camera.

### 2.7 PHB Extraction

Once observed its extra‐production in *Syn_*FMO, extraction of PHB was performed according to the protocol described by Cho et al. (2023). *Syn*_FMO strain was cultivated at OD_730_ = 1, in the absence or presence of 1 mM indole. Cells were transferred into Corex tubes and, after 24 h from the addition of the substrate, mixed with 75% chloroform then kept closed at 60°C to obtain phase separation. Finally, the organic phase was recovered and PHB film was obtained after total evaporation of residual chloroform.

### 2.8 Biotransformations Setup

Prior to biotransformations, 100 mL of *Syn_*FMO cultures at an OD_730_ = 0.1 were pre‐cultured in 300 mL flasks up to OD_730_ between 1 and 1.5. Then, cultures were harvested and resuspended in fresh BG11 to the desired working optical density. Whole‐cell biotransformations were performed in 30 mL Corning Corex tubes (5 mL working volume), under 200 rpm continuous shaking and constant light led illumination between 30 and 150 μmol photons •m^−2^ •s^−1^ by adding the substrate at chosen concentration (time 0). At fixed times, cultures were processed for indigo quantification.

### 2.9 Analytical Method for Indigo Quantification

Indigo content quantification was performed following an optimised protocol based on the separation of chlorophylls and indigo in two distinct steps (Figure S2). Samples were centrifuged for 8 min at 6000 g and 4°C. The supernatant was discarded and cells were resuspended in 90% methanol (by first adding water, 1/10 culture volume, and then methanol). After 30 min of incubation in darkness, tubes were centrifuged for 8 min at 6000 g and 4°C to separate the solubilised chlorophylls. Pellets were resuspended in DMSO and then centrifuged for 8 min at 6000 g at room temperature to separate cell debris. Indigo content of the samples was spectrophotometrically quantified at 620 nm after having produced a calibration line for each experiment (using samples of indigo dissolved in DMSO, from 0.625 mg/L to 10 mg/L). Measurements for both calibrating standard solutions and biotransformation extracts were performed in triplicate.

Specific activity (U/g_dcw_) and rate (mM/h) were calculated evaluating product appearance in the first 2 h after the addition of 1 mM indole to 0.5 mL culture. Reactions took place in 2 mL vials using cells at OD_750_ = 4. An OD_750_ = 1 corresponds to 0.24 g ^−1^L DCW.

### 2.10 Indigo Chemical Reduction

Indigo reduction was carried out by submerging the adsorbent in 40 mg/mL NaOH and 100 mg/mL Na_2_S_2_O_4_. After mixing, the final solution was heated at 60°C for 5 min, promoting the reduction of indigo.

## 3 Results and Discussion

### 3.1 Engineering and Molecular Characterisation of the Transgenic Strain *Syn*_FMO

A stable, transgenic *Synechocystis* strain expressing mFMO was generated by transforming the wild‐type strain with the plasmid pSuperP_FMO (Figure S1). In the transgene, transcription of the heterologous sequence was controlled by the well characterised *Synechocystis* ‘SuperPromoter’ P_cpc560_ (Zhou et al. 2014), a native promoter frequently exploited for overexpressing recombinant proteins in the cyanobacterium, and the terminator T_rbc_ (Liu and Pakrasi 2018). Notably, during the cloning and screening steps, *E. coli* colonies containing the final vector pSuperP_FMO appeared dark coloured already on LB agar plates. Since in *E. coli* cells the endogenous tryptophanase (TnaA) produces indole from tryptophan, the accumulation of indigo clearly indicated that the cyanobacterial promoter P_cpc560_ was at least partially functional in *E. coli* , and that the translated mFMO catalysed the oxidation of endogenous indole to indigo. This observation is of particular interest, since many bio‐bricks from *E. coli* do not function in *Synechocystis* (Liu and Pakrasi 2018; Opel et al. 2022).

The final strain was named *Syn*_FMO, and its homoplasmy was verified by PCR amplification (Figure S3). Moreover, the entire engineered insert, along with adjacent regions, was sequenced to ensure that no undesired mutation in the flanking psaK (ssr0390) and ndhB (sll0223) loci had occurred throughout the in vivo recombination (locus ssl0410; Pinto et al. 2015). In parallel, starting from the empty plasmid pSuperP560_UV (Figure S1), a ‘pseudo’ wild‐type strain called *Syn*_UV was produced and then used as an additional control strain in experiments aimed at monitoring cyanobacterial growth. Its phenotype, virtually identical to the wild‐type strain, demonstrated once again the absolute tolerance of the locus ssl0410 to knock‐out (Figure S4A and Table S2). Finally, expression of the mFMO with a C‐terminal His‐tag in *Syn*_FMO was verified by Western blotting (Figure S5).

### 3.2 Monitoring the Growth Phenotype of the Transgenic Strain *Syn*_FMO

**FIGURE 2:**
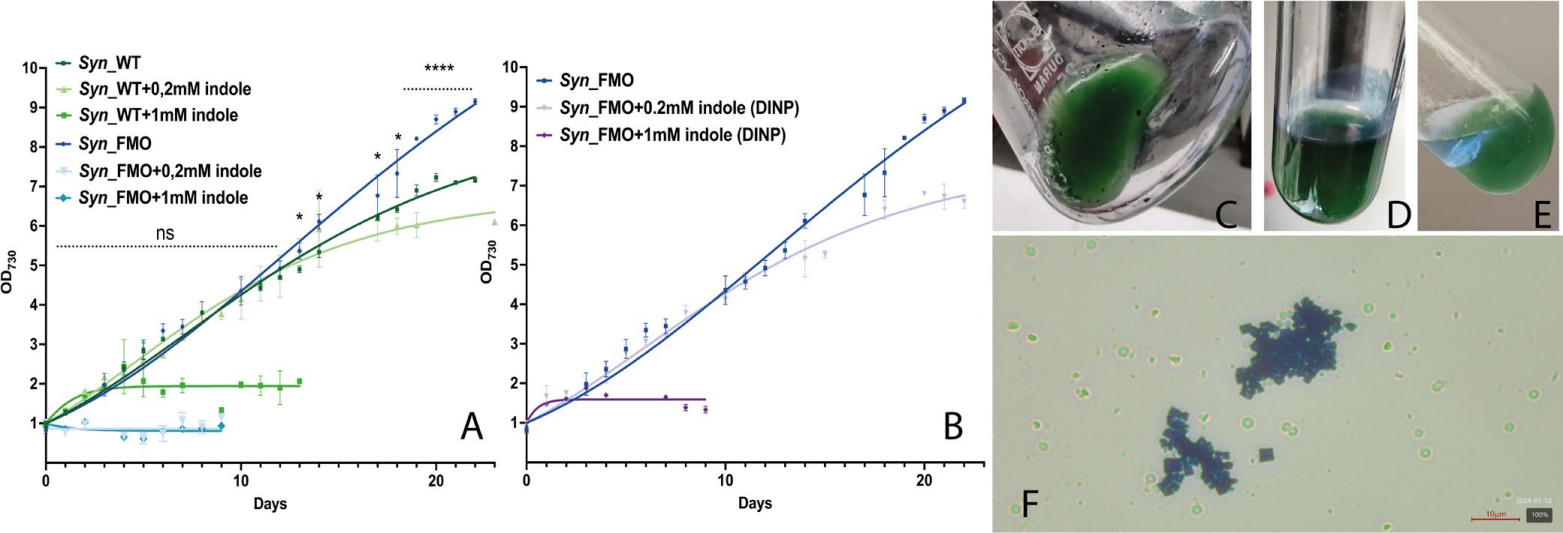
Growth curves and indigo secretion. (A) Curves comparing growth rates of wild‐type and *Syn*_FMO strains in the presence of different indole concentrations. Statistical significance (two sample t‐test) is indicated by ‘ns’ (not significant) or asterisks (significant, *p < 0.05 and ****p < 0.001) and is calculated for each time‐point measurement of *Syn*_WT and *Syn*_FMO without indole. (B) Curves comparing growth of strain *Syn*_FMO without indole, with 0.2 mM or 1.0 mM indole and a layer of the organic solvent DINP deposited on top of the growth medium. Cultures (three independent replicates for each strain and each condition) were cultivated in flasks under continuous shaking and constant light of 50 μmol photons •m^−2^ •s^−1^. (C–E) Examples of *Syn*_FMO cultures grown in the presence of indole in flask (C), Corex tube (D) and Corex tube with added DINP (E). (F) Drop of culture from D observed at the optical microscope (scale bar in red, 10 μM).

To assess the effects of mFMO expression on growth profile, we first examined growth curves for the *Syn*_FMO, wild‐type and *Syn_*UV strains over a time lapse of 20 days (Figures 2A and S4; Tables S2 and S3). The transgenic strain expressing mFMO showed higher biomass accumulation compared to the control strains. Moreover, as confirmed by cell counting (Figure S4A), *Syn_*FMO displayed continuous cellular division throughout the total cultivation. This phenotype is known for other NADPH‐dependent oxidoreductases expressed in recombinant form in *Synechocystis* (a useful list of examples is found in the review by Malihan‐Yap et al. 2022) and is explained by NADPH consumption due to the enzyme's activity, which leads to increased photosynthetic efficiency as a response to unbalanced NADPH/ATP ratio (Zhou et al. 2016). This was considered a preliminary indication that the FMO was active when expressed in the cyanobacterium, suggesting that the enzyme is consuming extra NADPH to promiscuously catalyse the oxidation of an endogenous molecule.

We then tested the growth of the *Syn*_FMO strain upon addition of different concentrations of indole in the culture medium (Figure 2A). Indole is well‐known to have toxic effects on the metabolism of bacteria (Kim et al. 2013), and such effects were observed in *Synechocystis* too.

Figure 2A shows that the presence and activity of the recombinant FMO abolishes growth of the *Syn*_FMO strain in the presence of indole, even at the lowest concentration tested (0.2 mM), which instead allows growth of the control strain. Indeed, after only 24 h, flasks containing the FMO‐expressing strain and indole ranging from 0.2 to 1.0 mM clearly exhibited the accumulation of dark insoluble clots in the growth medium (Figure 2C) or the formation of a blue stratus on the glass surface of the vessel (Figure 2D). These clots, observed closely by optical microscopy (Figure 2F), were shown to consist of crystalline indigo aggregates (see forward).

To determine which of the molecules, substrate or product, was more detrimental to the growth, we monitored cellular proliferation for 7 days in the presence of added 0.5 mM indigo (corresponding to 100% conversion of 1 mM indole, Figure S4B). In contrast to indole, indigo did not negatively affect the growth of either the wild‐type or *Syn_*FMO strains.

We also tested the growth of *Syn_*FMO in BG11 medium supplemented with indole (0.2 mM and 1 mM) in the presence of a small aliquot (1–2 mL) of the water‐immiscible diisononyl phthalate (DINP) layered over the culture. This organic liquid phase was already shown to be compatible with different recombinant strains of *Synechocystis* employed in whole‐cell biotransformations (Hoschek et al. 2019a, 2019b). DINP was effective in recovering the growth of the transgenic strain, similar to the control (Figure 2B). Since both indole and indigo are likely to be soluble in DINP, the organic phase was effective in reducing the concentration of both molecules in the medium, thus allowing cellular proliferation. This observation, at least for indigo, was confirmed by the formation of a blue colour in the DINP layer (Figure 2E). The blue colour was rapidly lost, confirming that the dye is unstable in the phthalate (Mocquard et al. 2022).

### 3.3 Transmission Electron Microscopy Revealed PHB Overproduction in *Syn_*FMO

**FIGURE 3:**
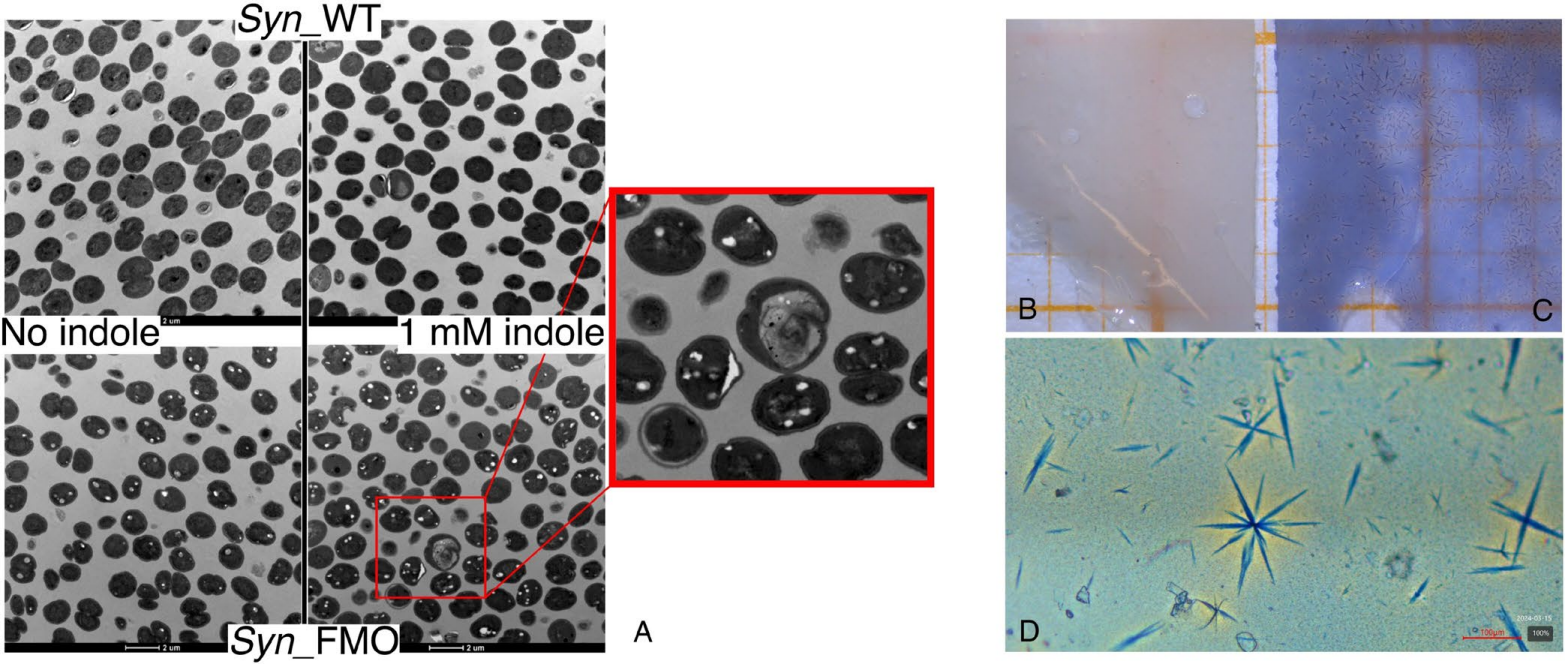
TEM images and PHB extraction from *Synechocystis* cells. (A) Transmission electron micrographs of wild‐type *Synechocystis* and *Syn*_FMO cells cultured in standard conditions (constant light of 50 μmol photons •m^−2^ •s^−1^) in the absence and in the presence of 1 mM indole concentrations. On the right, enlarged particular of the inset framed in red. Scale bars (2 μm) as indicated in individual micrographs. (B–D) Above, stereo microscope images showing surface morphology of polyhydroxybutyrate (PHB) films obtained from extraction and drying of the polymer from Syn_FMO cells, cultivated in standard conditions (constant light of 50 μmol photons •m^−2^ •s^−1^) without (B) or with (C) 1.0 mM indole. Film fragments were placed on a graph paper (visible yellow grid, side of a square corresponds to 1 mm). Below, (D) optical microscope images of the blue coloured PHB film, scale bar in red (100 μm).

Given the observed toxicity of indole and considering its hydrophobic nature, we sought to investigate its harmful effects, particularly at the level of cellular membranes. Samples from wild‐type *Synechocystis* and *Syn*_FMO cultures in the early logarithmic phase of growth were analysed by transmission electron microscopy (TEM) 24 h after the addition of 0.2 mM and 1.0 mM indole. Interestingly, TEM analysis revealed the presence of electron‐transparent spots exclusively in the transgenic strain *Syn*_FMO, both in the absence and presence of indole (Figure 3A). Similar spherical inclusions are generally assumed to be polyhydroxybutyrate (PHB) granules.

*Synechocystis* is a natural PHB producer, which is synthesised in the late stages of nutrient, oxygen or light stress (Koch et al. 2019). Natural or engineered PHB overproducer mutant strains are actually being investigated as new sources of bioplastics (Koch et al. 2020; Grivalský et al. 2024). The biosynthetic pathway of PHB starts from acetyl‐CoA, a central molecule in the general lipid metabolism, including the tricarboxylic acid cycles, as well as the synthesis of fatty acids, ethanol and alkanes. It is therefore plausible that the mere presence of the active FMO, which modifies an unknown endogenous molecule in the absence of indole while consuming NADPH (as previously speculated), could cause the unbalancing of some reaction that, in turn, causes cellular stress and the appearance of PHB (Eungrasamee et al. 2024).

TEM images also revealed that, 24 h after the addition of indole, *Syn*_FMO cells displayed considerable morphological features consistent with cell stress and toxicity (Figure 3A), with a higher number of white spots (an average of 1.20 spot/cell at 1.0 mM indole compared to 0.79 spot/cell without indole; see Table S4 for statistical analysis). This confirms that indole toxicity is enhanced in *Syn_*FMO. In such burdensome stress conditions, fractures of the cell envelope with extracellular leakage of PHB granules were also observed (see magnification in Figure 3A). The number of fractured cells was greater in the culture containing 1.0 mM indole compared to those with no or 0.2 mM indole. We suggest that all the molecules involved in the cascade reaction (particularly indole; Figure 1) might contribute to the weakening of the cell membranes due to their hydrophobic nature. Moreover, the accumulation of crystalline indigo could further increase membrane fragility, as observed in *E. coli* cells (Han et al. 2013). Considering the wide and continuous network of internal membranes (i.e., thylakoids, where the vital processes of photosynthesis take place) inside the cyanobacterial cell, the higher toxicity of indole in *Synechocystis* compared to *E. coli* (tolerating up to 5 mM indole; Chu et al. 2012) is easily explained.

One‐pot production of indigo and PHB in recombinant *E. coli* was already explored for producing coloured bioplastic (Jung et al. 2020; Cho et al. 2023). The intriguing results of the TEM analysis prompted us to check the competence of the cellular PHB for adsorbing the indigo dye produced in *Syn*_FMO. Given that in *Synechocystis* we had observed secretion of indigo and, upon cell breakage, the release of PHB in the medium, we adapted our biotransformation protocols to produce plastic films, as described by Jung et al. (2020). Gratifyingly, films obtained from *Syn*_FMO grown for 24 h in the presence of 1.0 mM indole were blue‐coloured (Figure 3C), in contrast to those obtained in the absence of indole (Figure 3B). When observed using optical microscopy, the blue PHB‐films showed indigo crystals formed throughout the process of extraction and drying of the polymer (Figure 3D). These results clearly indicate that the PHB granules observed in our TEM analysis may function in capturing and buffering at least part of the blue dye produced via the indigo‐forming enzyme.

### 3.4 Setting Up Biotransformations

Initial biotransformations were performed in 100 mL flasks or Corex tubes. The latter proved particularly useful for developing an extraction and solubilisation protocol that enabled the quantification of indigo, cleared of chlorophyll (Experimental Procedures and Figure S2). Dimethyl sulfoxide (DMSO), the standard solvent used for measuring indigo produced by fermentation of recombinant bacteria, could not be directly used with *Synechocystis* culture samples, as it also extracts pigments (essentially chlorophyll) from the cyanobacterial membranes.

**FIGURE 4:**
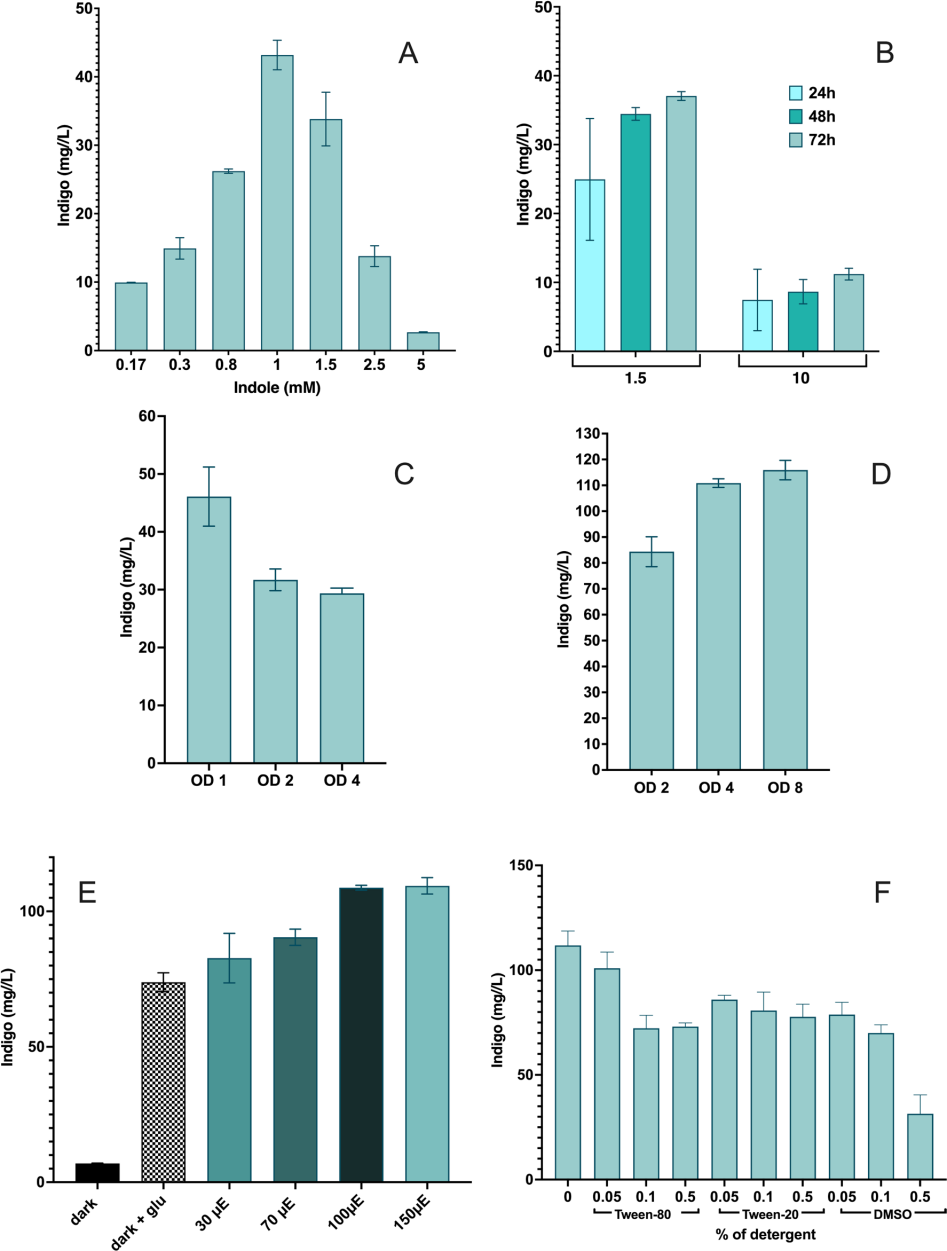
Optimisation of indigo production. Indigo yields of biotransformations performed in different conditions. Histograms present data on (A) indigo production by *Syn*_FMO cultures grown for 72 h at various concentrations of indole; (B) production over times in the presence of two different concentrations of indole; (C) yields at 72 h in biotransformations performed in 0.5 mM indole, starting from *Syn*_FMO cultures grown up to different ODs; (D) yields obtained (at 72 h, 1 mM indole) by *Syn*_FMO cells grown up to OD_730_ = 1 then concentrated at different ODs, under high light illumination (150 μmol photons •m^−2^ •s^−1^); (E) Indigo yield at 72 h in biotransformations with 1.0 mM indigo, cells from early growth phase concentrated at OD_730_ = 4, in different conditions of illumination (expressed in μE = μmol photons •m^−2^ •s^−1^) and growth medium (glu = 5 mM glucose). (F) Indigo yield at 72 h in biotransformations with 1.0 mM indigo, cells from early growth phase concentrated at OD_730_ = 4, under 100 μmol photons •m^−2^ •s^−1^ and growth medium supplemented with different detergents at different percentage (from 0% to 0.5%). Three independent replicates for each experiment were analysed.

Subsequently, we explored the best conditions for the biotransformation at a relatively small scale (Figure 4 and Supporting Information). We performed experiments to determine: (i) highest tolerated concentration of indole assuring highest yield of indigo (Figure 4A,B); (ii) the preferred growth phase for expression of mFMO from the P_cpc560_ promoter (Figure 4C); (iii) the ideal working cell density (Figure 4D) and (iv) the dependence of yield on light intensity (Figure 4E). We consequently established 1.0 mM indole and cells from the early phase of growth (OD_730_ = 1) concentrated to OD_730_ = 4 as preferable conditions for carrying out biotransformations. As for the last parameter, light intensity, it emerged as critical for two reasons. A high light intensity is necessary to sustain a productive photosynthetic process and mitigate the self‐shading effect of growing cells, which is made worse by the filter effect due to the accumulation of indigo in the broth. Conversely, excessive illumination triggers physiological photoprotective processes that counteract damages to photosystems and ultimately reduce photosynthesis (Ogawa et al. 2018). Therefore, since the foremost aim of this work was to produce indigo by only exploiting photosynthesis, we evaluated biotransformations under both high and low light intensities, i.e., abundant and restricted provision of NADPH respectively. As a matter of fact, it has been shown that, in some biotransformations, the availability of cofactors can limit the reactions (Assil‐Companioni et al. 2020; Mascia et al. 2022). Biotransformations of 1.0 mM indole and (final) OD_730_ = 4 were tested under different light intensities (30, 70, 100 and 150 μmol photons •m^−2^ •s^−1^). As a proof of light dependence, biotransformations were also tested in darkness, with and without an added organic carbon source (5 mM glucose) (Figure 4E).

In the range of light intensities tested, photosynthesis effectively supported FMO activity, with a progressive increase in indigo production as illumination increased. The conversion reached 62% at 30 μmol photons •m^−2^ •s^−1^, 68% at 70 μmol photons •m^−2^ •s^−1^, and peaked at 85% at both 100 and 150 μmol photons •m^−2^ •s^−1^. As previously stated, the main limiting effect observed in this whole‐cell biotransformation was the one ascribable to the toxicity of the molecules involved in the reaction. Under heterotrophic conditions, indigo production was lower than that observed under phototrophic conditions at the lowest light intensity, confirming that NADPH regeneration mainly relies on the photosynthetic process.

Under the best identified conditions, the maximum titre of indigo obtained was 112 mg/L; the maximum rate of the reaction, calculated at the initial phase, was 0.056 ± 0.003 mM·h^−1^, and cell specific activity was 0.98 ± 0.05 U/g_DCW_. This value is comparable to what has been measured for *Synechocystis* cells expressing other monooxygenases and employed in different biotransformations (Malihan‐Yap et al. 2022).

### 3.5 Bio‐Indigo Production Improvement and Recovery by Adsorbent Materials

Indigo is highly insoluble in water, while indole is very slightly soluble in water. These chemical properties hinder the exchanges between cells and growth medium, thereby enhancing the toxicity of the two molecules, as previously underlined. Aiming to facilitate indole diffusion inside the cell and enhance indigo secretion, Tween20, Tween80 and DMSO detergents were tested at concentrations of 0.05%, 0.1% and 0.5% in the culture medium. This approach had previously been used in *E. coli* cells expressing indole‐converting enzymes, such as mFMO or the toluene monooxygenase from *Pseudomonas mendocina* . In *E. coli* , 1% DMSO was effective in raising the indigo production by 17% with respect to the control biotransformation (Ham et al. 2023; Yuk et al. 2023). In contrast, our results indicated that the presence of any concentration of DMSO, Tween20 or Tween80 reduced indigo production (Figure 4F; see Table S5 for measured yields and calculated conversions). This outcome confirms the previously observed fragility of the cyanobacterial membrane.

**FIGURE 5:**
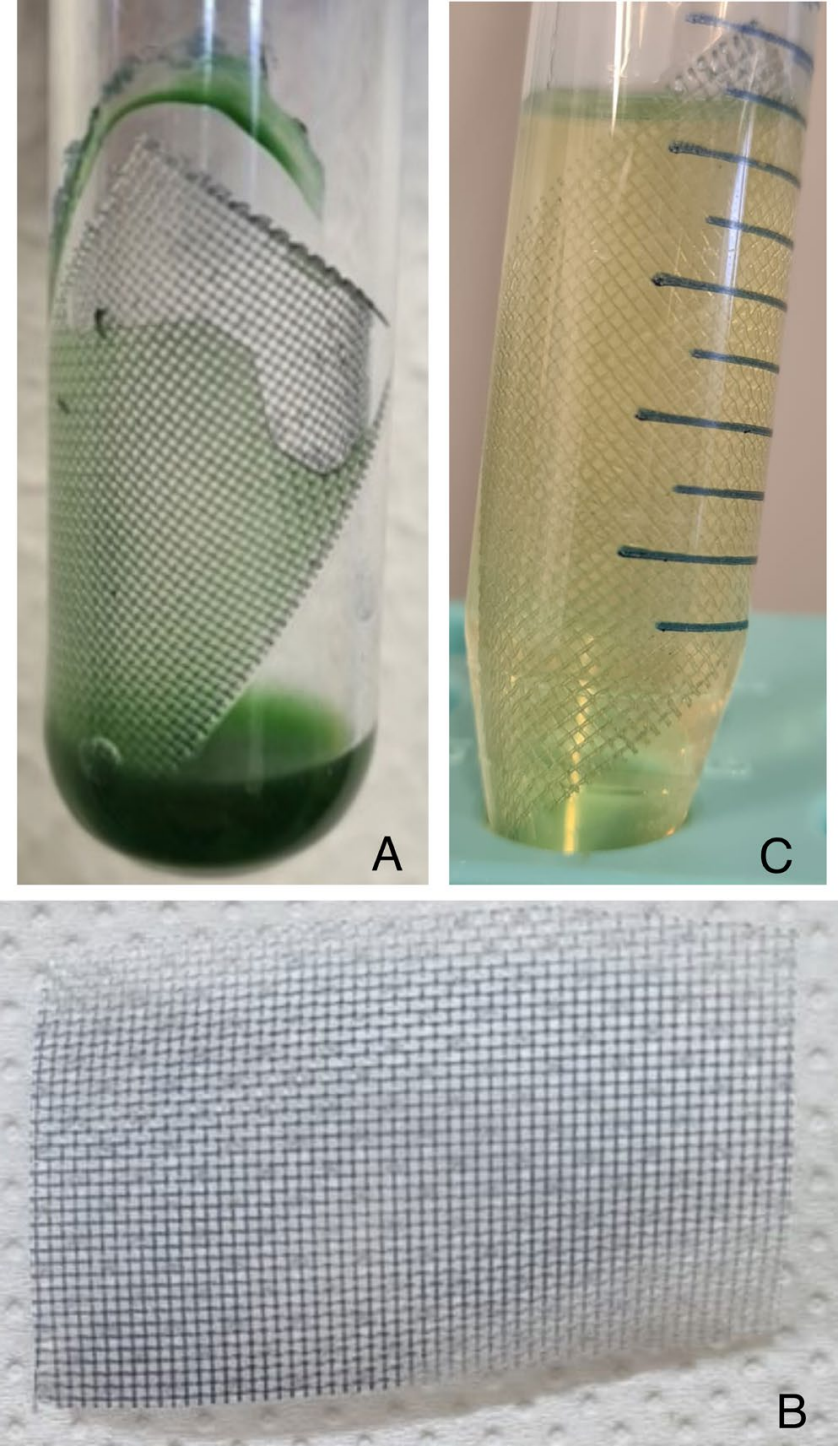
Indigo recovery from the growth medium. (A) *Syn_*FMO biotransformations performed with 1.0 mM indole, 150 μmol photons •m^−2^ •s^−1^ constant illumination after 72 h in the presence of an adsorbent polyamide netted film. (B) Polyamide netted film after biotransformation, washing and drying. (C) Reduction of indigo adsorbed by polyamide netted film (reduction mixture consisting in 40 mg/mL NaOH and 100 mg/mL Na_2_S_2_O_4_) with release of leuco‐indigo.

We then considered the direct recovery of secreted indigo by using hydrophobic materials directly inserted in the culture vessels. Such material might act as an in situ trap or sponge, efficiently extracting indigo from the medium. Three kinds of polymer materials were tested: styrene‐divinylbenzene beads, expanded polyethylene and polyamide nets (Table S6). The highest yield was obtained by polyamide netted films adherent to the inner surface of Corex tubes (Figure 5A). A total of 81 mg/L indigo was recovered attached to the film (71% of total produced indigo; Figure 5B). Having indigo attached to a solid material would represent an advantage for downstream processes and dyeing, that imply indigo reduction into its soluble leuco‐indigo form. Therefore, we investigated the reduction of indigo by treating the coloured polyamide nets with an adequate reducing mixture (see Section 2). As shown in Figure 5C, successful reduction was achieved. Furthermore, the polyamide nets could be re‐used for new cycles of indigo adsorption and reduction.

## 4 Conclusions

The premise to this study was to evaluate the ability of *Synechocystis* cells expressing mFMO to produce the popular indigo dye by light‐mediated biotransformation. The results of our research presented here show that the cyanobacterium endowed with mFMO, in the presence of light, is indeed a competent host for the synthesis of indigo. Although the yields are lower compared to those obtained in *E. coli* , mostly upon subsequent engineering steps, several advantages of using a transgenic, stable *Synechocystis* strain for bio‐indigo production become apparent. Among others, the advantage of avoiding the addition of an antibiotic all along the biotransformation; the saving of sugars as a carbon source while simultaneously consuming CO_2_; the secretion of the dye and its easy recovery from the growth medium, which enhance the overall efficiency and viability of downstream processes. The combination of these factors results in a high attractiveness and encourages further exploration to evolve and fine‐tune the biotransformation process, with the aim of making it feasible and convenient for a scaling‐up.

## Author Contributions

**Giovanni Loprete:** conceptualization, investigation, writing – original draft, methodology, formal analysis, data curation, validation. **David Rubert:** investigation, conceptualization. **Francesco Bellusci:** investigation. **Nikola Lončar:** writing – review and editing, resources. **Marco W. Fraaije:** writing – review and editing, resources. **Elisabetta Bergantino:** conceptualization, funding acquisition, methodology, formal analysis, supervision, writing – review and editing, resources.

## Conflicts of Interest

The authors declare no conflicts of interest.

## Supporting information


Appendix S1.

## Data Availability

The data that support the findings of this study are available from the corresponding author upon reasonable request.
