# Supplementary material for: Greening the Production of Indigo Blue Exploiting Light and a Recombinant Synechocystis sp. PCC6803 Strain Expressing the Enzyme mFMO
Source: Microb Biotechnol. 2025 Apr 28;18(5):e70146. doi: 10.1111/1751-7915.70146 (PMC12035870; doi:10.1111/1751-7915.70146)
Supplement: Supplementary file 1 — Appendix S1. [file MBT2-18-e70146-s001.pdf]

SUPPORTING INFORMATION to the article

Greening the production of indigo blue exploiting light and a recombinant *Synechocystis* sp. PCC6803 strain expressing the enzyme *m*FMO

Giovanni Loprete [a], David Rubert [a], Francesco Bellusci [a], Nikola Lončar [b], Marco W. Fraaije [c] and Elisabetta Bergantino *[a]

[a] Synthetic Biology and Biotechnology Unit, Department of Biology, University of Padova, Viale G. Colombo 3, I-35131 Padova, Italy

[b] Gecco Biotech, Zernikepark 6-8, 9747AN, Groningen, The Netherlands

[c] Molecular Enzymology Group, University of Groningen, Nijenborg 4, 9747AG Groningen, The Netherlands

CONTENTS

Supplementary Results

Setting optimal conditions for biotransformation.. 2

Supplementary Figures and Tables

Figure S1: Maps of plasmids pSuperP_UV and pSuperP_FMO 3

Figure S2: UV-Vis spectra of chlorophyll and indigo extracted from *Syn*_FMO cultures 4

Figure S3: Colony PCR amplification of the WT, *Syn*_UV and *Syn*_FMO strains 5

Figure S4: Growth curves drawn by measuring OD_{730} and cell-counting....................................... 6

Figure S5: Western-Blot analysis of *Syn*_FMO protein extract 7

Table S1: List of oligonucleotides used in this study.. 8

Table S2: Results of analysis of non-linear fitting regression (Fig. S4, A and B).......................... 9

Table S3: Results of analysis of non-linear fitting regression (Fig. 2, A and B).......................... 10

Table S4: Statistical analysis of TEM images 11

Table S5: Yields of biotransformations supplemented with detergents 12

Table S6: Yields of indigo recovered by different adsorbents... 12

Supplementary References 13

Supplementary Results

Setting optimal conditions for biotransformation

To the aim of identifying the best concentration of indole assuring lowest toxicity and highest production of indigo, cultures were grown in standard conditions to OD_{730}=2, then different concentrations of the substrate were added (Fig. 4, A). Quantification was performed at 72 hours after addition of indole, i.e. the time at which the maximum yield was observed (Fig. 4, B). Consequently, we chose the concentration of the substrate around 1 mM for the following experiments of whole-cell biotransformations.

We then moved to determine the best condition of OD_{730} to maximize the yield of the biotransformation. It has to be underlined here that the Cpc560 promoter (P_{cpc560}, that drives the transcription of the FMO gene in the transgenic strain) is the native promoter of the gene encoding the ß subunit of c-phycocyanin (cpcB), a pigment-binding protein that is one of the most abundant in cyanobacteria (Zhou et al., 2014). Integrative analysis of *Synechocystis* transcriptome revealed that the transcription of the cpcB gene is affected by the growth phase (Singh et al., 2010). Moreover, fluorescence measurements of enhanced-Yellow Fluorescent Protein (eYFP) under the control of P_{cpc560} showed that protein expression is higher during the early growth phase while is reduced during the stationary phase (Ng et al., 2015). Therefore, with the aim to assess the impact of the growth phase on FMO expression and indigo production, biotransformations were performed starting from cultures at OD_{730} of 1, 2 and 4. Cultures were grown in standard conditions and once reached the desired OD, cells were concentrated or diluted to a final OD_{730}= 2.

According to the article cited above, the highest activity of the FMO enzyme, corresponding to the highest yield of indigo, was obtained in the early growth phase (OD_{730}=1). Fig. 4, C shows that, as the growth phase increases, indigo production decreases.

Therefore, after having set at 1 mM the concentration of substrate indole for biotransformations, we opted to perform the reactions starting from cultures grown up to OD_{730}= 1. Consequently, the *Syn*_FMO cultures were grown to OD_{730}= 1 and then concentrated to OD_{730} 2, 4, and 8 for testing biotransformations at high light intensity (150 µmol photons •m^{-2} •s^{-1}), to the aim of evaluating a possible self-shading effect due to high concentration of cells in the culture. It is known, in fact, that such effect rises at high optical density (around OD_{730}=10) and leads to a decrease of the overall photosynthetic process efficiency (Böhmer et al., 2017) with consequent lowered regeneration of NADPH. Fig. 4, D shows the trend of indigo production as registered in this test: the production increased from 65% (OD_{730}=2) to 85% (OD_{730}=4) and reached its maximum already at OD_{730}=8 (87%). However, we observed that the increase in indigo yield between OD_{730} 4 and 8 was not worth for doubling the cell density. We interpreted the slowing-down in production as the result of two overlapping factors: high indigo toxicity and self-shading effect, the latter factor being emphasized by the presence of the blue dye both in the growth medium and inside the cell. We therefore set to concentrate

cells from the early phase of growth (O.D.$_{730}$=1) to O.D.$_{730}$=4 as preferable condition for carrying out biotransformations.

Supplementary figures and tables

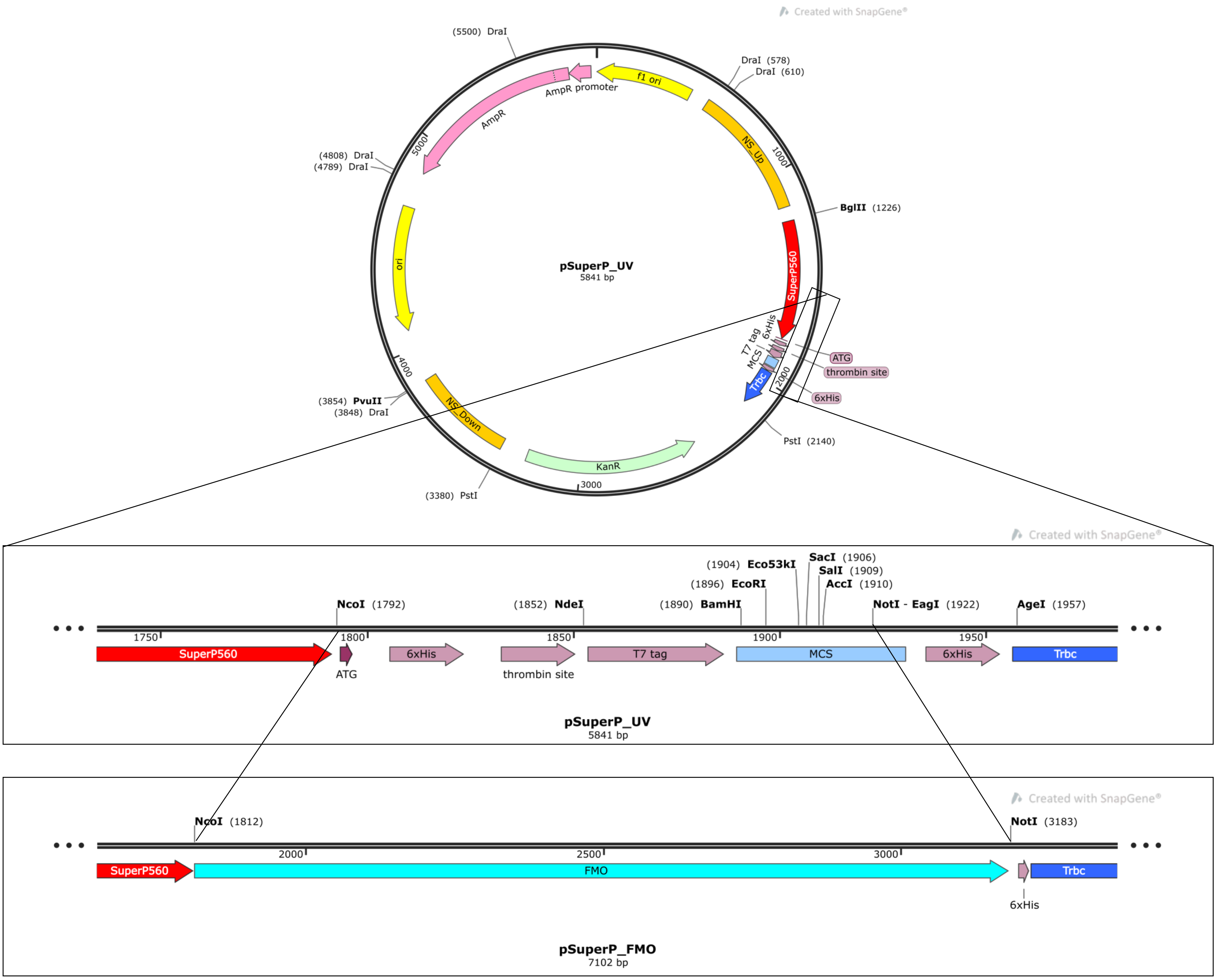

Fig. S1. Maps of plasmids pSuperP_UV and pSuperP_FMO. Maps obtained by the SnapGene® software (from Dotmatics; available at snapgene.com).

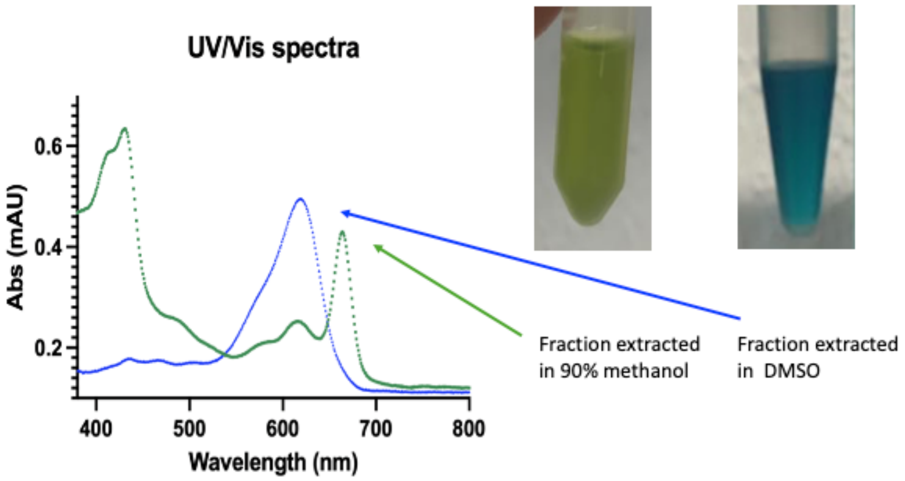

Fig. S2. UV-Vis spectra of chlorophyll and indigo fractions extracted from *Syn*_FMO cells. The green curve corresponds to the spectrum of the first fraction extracted, by using 90% methanol, (containing chlorophyll and carotenoids). In blue, spectrum registered for the second fraction, obtained by subsequent extraction using DMSO, showing the characteristic absorption peak of indigo at the wavelength of 620 nm.

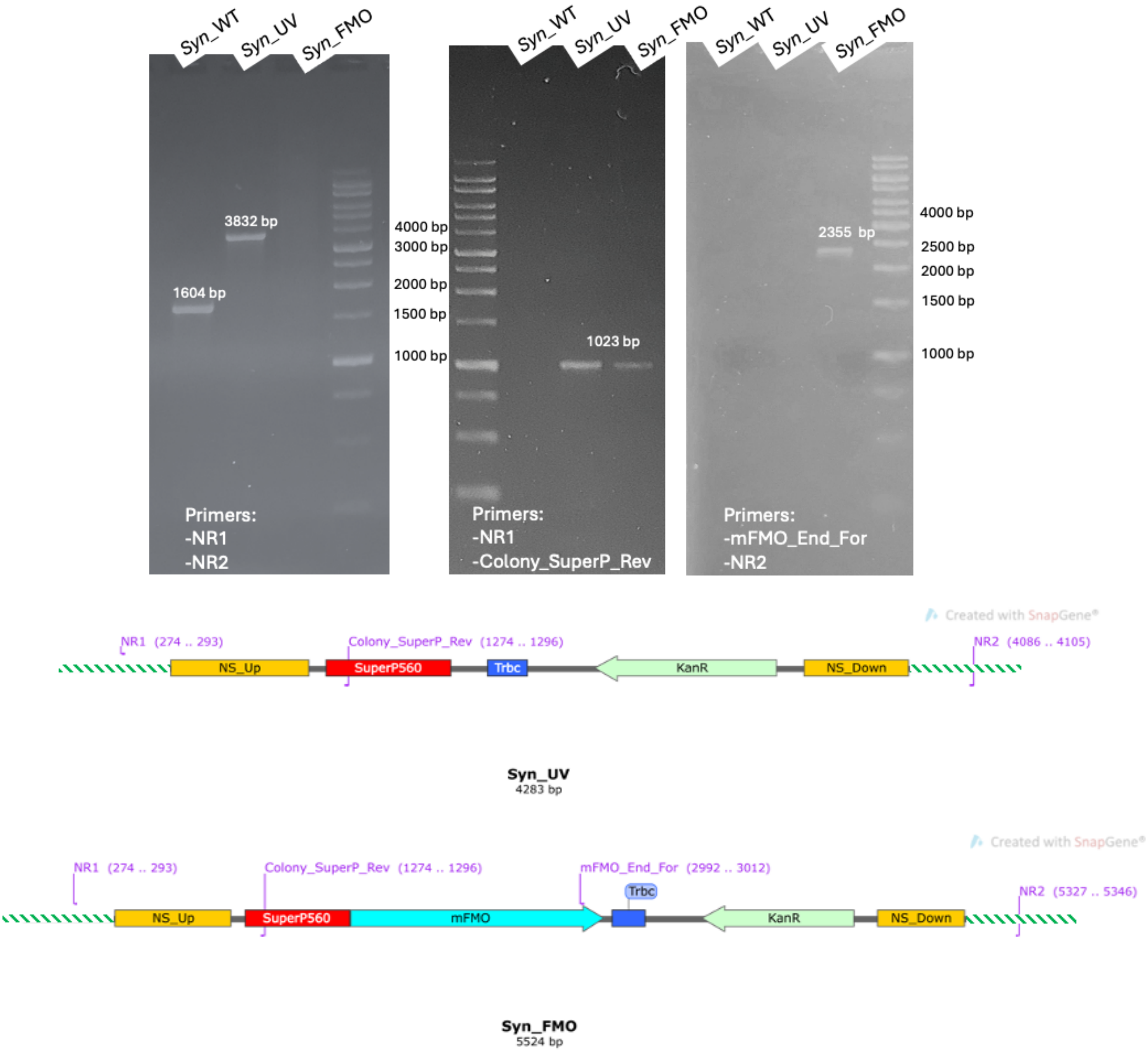

Fig. S3. Agarose gel analysis of products obtained by colony PCR amplification of the wild-type, the “pseudo” wild-type *Syn*_UV and *Syn*_FMO strains. Colony PCR amplifications were used for verifying (*i*) correct insertion of the engineered sequence and (*ii*) achievement of homoplasmy in the transgenic cells. Scheme below the image shows the map of the chromosomic region of interest, as expected after transformation and recombination; positions of primers employed for amplifications are shown; green dashed segments represent regions of the cyanobacterial chromosome flanking the neutral site chosen for insertion (Experimental section of the main manuscript).

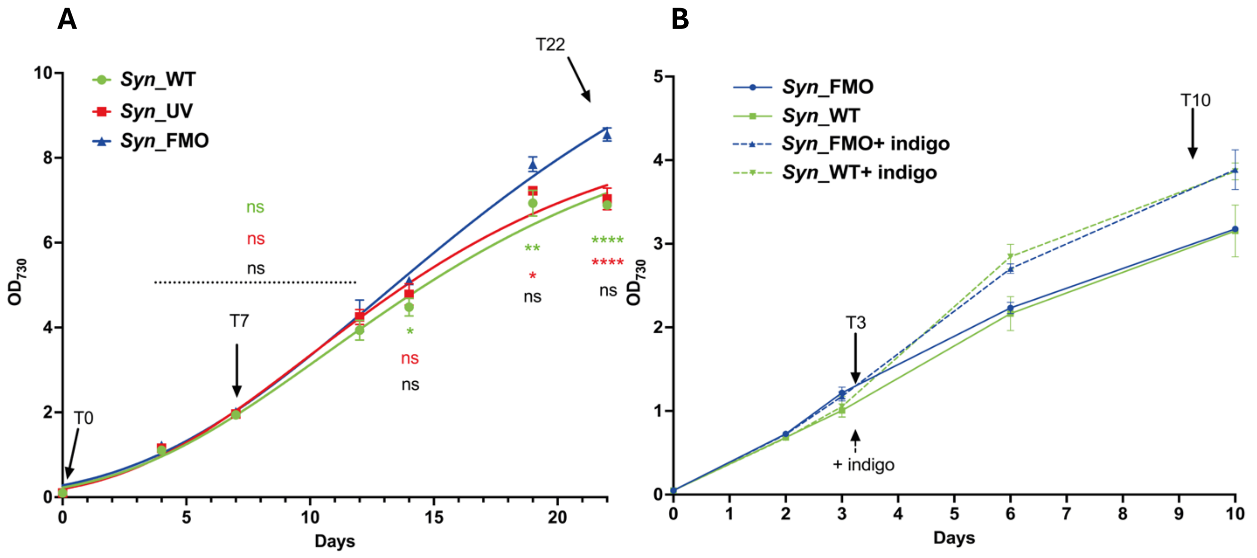

	*Syn*_WT	*Syn*_UV	*Syn*_FMO
**T0 (*10^8 cells/mL)**	0.08±0.01	0.08±0.02	0.08±0.01
**T7 (*10^8 cells/mL)**	1.92±0.12	2.04±0.08	1.94±0.13
**T22 (*10^8 cells/mL)**	10.31±0.76	9.33±0.75	15.03±1.65

	*Syn*_FMO	*Syn*_WT	*Syn*_FMO +indigo	*Syn*_WT +indigo
**T3 (*10^7 cells/mL)**	3.21±0.17	3.22±0.14	3.18±0.15	3.19±0.18
**T10 (*10^7 cells/mL)**	27.21±1.93	25.2±2.21	30.3±5.34	33.8±0.92

Fig. S4. Growth curves drawn by measuring OD_{730} and cell counting. (**A**) Curves comparing growth profiles of *Syn*_FMO, wild-type and *Syn*_UV strains in the in absence of indole together with cell counts measured at day 0, day 7 and day 22. Statistical significance (One-way ANOVA test) is represented by "ns" (not significant) or asterisks (significant) and is calculated for each time-point measurement. Significance between *Syn*_FMO and *Syn*_WT, as well as *Syn*_FMO and *Syn*_UV, is represented in green and red, respectively, while significance between *Syn*_WT and *Syn*_UV is represented in black. (**B**) Curves comparing growth profiles of *Syn*_FMO and wild-type in the absence and presence of 0.5 mM indigo together with cell counts measured before and 7 days after indigo addition.

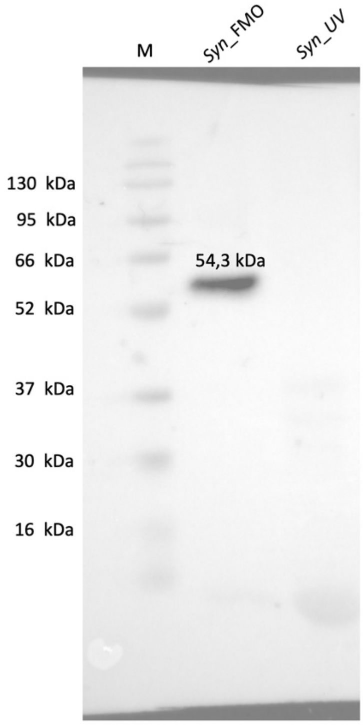

Fig. S5. Western-Blot analysis of *Syn*_FMO and Syn_UV protein extracts. Total protein extracts of Syn_FMO and Syn_UV cells cultured in BG11 medium and standard conditions for growth. Immunodetection by anti His-Tag-HRP antibody (SB194b, Southern Blotting, Birmingham, USA). The indicated molecular weight of FMO is calculated from the primary sequence of the His-tagged protein.

Oligo name	Sequence 5'→3' *	Purpose
Pcpc560_for	GGCCAGATCATCGGGTAGAC	Pcpc560 *Synechocystis* PCC6803 genomic amplification
Pcpc560_rev	TGGAAACGATAGCGGAAGC	
Trbc_for	GTACTTTGGGTCACCCCTGG	Trbc *Synechocystis* PCC6803 genomic amplification
Trbc_rev	TCCGTTGGAGGTTTCCGAAG	
SuperP_BglII_for	TATATATAAGATCTACCTGTAGAGAAGAGTCCCTGAA	Cloning of Pcpc560 in pET28a(+) plasmid, inserts BglII site
SuperP_NcoI_rev	TATATATACCATGGTGAATTAATCTCCTACTTGACTTTATG	Cloning of Pcpc560 in pET28a(+) plasmid, inserts NcoI site
HistagRbcTer_for	TATACTCGAG*CACCACCACCACCACCAC*TGAACCGGTGTTTGGATTGTCGG	Cloning of Trbc in pET28a(+) plasmid, inserts XhoI site
RbcTer_rev	TATACTCGAGCTGCAGGCTGTCGAAGTTGAACATCAG	Cloning of Trbc in pET28a(+) plasmid, inserts PstI and XhoI sites
NR1	TGGCAAATTCAAGGGGTGGT	External NS region amplification
NR2	TTACCGATGAGTGGCACGTT	
PvuII_NS1_For	ATATATCAGCTGAACGGTGGTTTCCCAGGGG	NS1 fragment forward
NS1_mut_Rev	TCAGGAAATATTGCTCTGCAGGCA	Mutagenic primer, inserts PstI site, overlaps extension fragment 1
NS2_mut_For	TGCCTGCAGAGCAATATTTCCTGA	Mutagenic primer, inserts PstI site, overlaps extension fragment 2
DraI_PvuII_NS2_Rev	ATATATCAGCTGTTTAAAGACCCCTTGTTAACCTTGG	NS2 reverse
FMO_For_NcoI	ATCCTAATCCATGGCAACCCGTATCG	Mutagenic primer, inserts NcoI site
FMO_Rev_NotI	ATGCGGCCGCTGCTTCTTTCGC	Mutagenic primer, inserts NotI site
Colony_SuperP_Rev	AGTGGGGTAGGGAAGACTGTTGC	Pcpc560 amplification
mFMO_End_For	GTGATCATAGCTATCGCTCTC	mFMO amplification

Table S1. List of oligonucleotides used in this study

* Restriction sites inserted by mutagenic primers are underlined; His-tag coding sequence in italic letters.

	***Syn*_WT**	***Syn*_UV**	***Syn*_FMO**
**Gompertz growth Best-fit values**			
YM	9,154	8,909	12,76
Y0	0,2274	0,1950	0,2794
K	0,1235	0,1361	0,1046
1/K	8,099	7,348	9,557
**95% CI (profile likelihood)**			
YM	8,035 to 11,05	7,954 to 10.43	11,21 to 15,20
Y0	0,09414 to 0,4145	0,07034 to 0,3833	0,1636 to 0,4221
K	0,09611 to 0,1538	0,1071 to 0,1686	0,08662 to 0,1238
1/K	6,504 to 10,40	5,930 to 9,337	8,076 to 11,54
**Goodness of Fit**			
Degrees of Freedom	18	18	18
R squared	0,9870	0,9866	0,9945
Sum of Squares	1,716	1,910	1,052
Sy.x	0,3087	0,3257	0,2418

Tab. S2. Results of analysis of non-linear fitting regression (Gomperz growth) of growth curves presented in Fig. S4, A and B. Data were obtained by GraphPad Prism software.

	***Syn*_WT**	***Syn*_WT +0,2mM**	***Syn*_WT +1mM**	***Syn*_FMO**	***Syn*_FMO +0.2mM**	***Syn*_FMO +1mM**
**Gompertz growth Best-fit values**						
YM	8,57	6,776	1,834	14,34	0,86	0,8054
Y0	= 1,000	= 1,000	= 1,000	= 1,000	= 1,000	= 1,000
K	0,1117	0,1467	1,076	0,08018	Unstable	0,7038
1/K	8,952	6,816	0,9293	12,47	Unstable	1,421
**95% CI (profile likelihood)**						
YM	8,201 to 9,447	6,365 to 7,263	1,716 to 1,953	12,40 to 17,29	0,7522 to 0,9678	0,4838 to 0,8920
K	0,1014 to 0,1226	0,1301 to 0,1653	0,5713 to 5,020	0,06947 to 0,09137	(Very wide)	0,05239 to ---
1/K	8,158 to 9,860	6,050 to 7,688	0,1992 to 1,750	10,94 to 14,39	(Very wide)	--
**Goodness of Fit**						
Degrees of Freedom	32	35	34	32	16	16
R squared	0,9885	0,9605	0,3225	0,9812	0,05005	0,0769
Sum of Squares	1,837	4,724	3,434	4,129	0,6614	0,3135
Sy.x	0,2396	0,3674	0,3178	0,3592	0,2033	0,14

Tab. S3. Results of analysis of non-linear fitting regression (Gomperz growth) of growth curves presented in Fig. 2, A and B. Data were obtained by GraphPad Prism software.

**Strain & Indole concentration**	**Nr. Spots/cell**
**(x1) Syn_WT + 0 mM**	0,03 ±0,02
**(x2) Syn_WT + 0,2 mM**	0,06±0,01
**(x3) Syn_WT + 1 mM**	0,21 ± 0,03
**(x4) Syn_FMO + 0 mM**	0,79 ± 0,11
**(x5) Syn_FMO + 0,2 mM**	0,92 ± 0,05
**(x6) Syn_FMO + 1 mM**	1,20 ± 0,04

**Pair**	**Difference**	**p-value**
**x1-x2**	0.03268	0.9437
**x1-x3**	0.1776	0.001609
**x1-x4**	0.7583	1.145e-12
**x1-x5**	0.8845	1.143e-12
**x1-x6**	1.1737	1.143e-12
**x2-x3**	0.1449	0.01197
**x2-x4**	0.7256	1.15e-12
**x2-x5**	0.8518	1.143e-12
**x2-x6**	1.1411	1.143e-12
**x3-x4**	0.5807	4.418e-12
**x3-x5**	0.7069	1.194e-12
**x3-x6**	0.9961	1.143e-12
**x4-x5**	0.1262	0.02302
**x4-x6**	0.4154	8.991e-10
**x5-x6**	0.2892	6.994e-7
**Total D of freedom**	**F-Stat**	**p-value**
28	365.8776	0

Table S4. Statistical analysis of TEM images. Five independent micrographs were taken in account for the analysis. On the left, table containing average and standard deviation. On the right, report of One-way ANOVA Test.

	**no additives**	**Tween-80**			**Tween20**			**DMSO**		
		**0,05%**	**0,1%**	**0,5%**	**0,05%**	**0,1%**	**0,5%**	**0,05%**	**0,1%**	**0,5%**
**Yield (mg/L)**	111,74 ± 6,83	106,79 ± 11,58	72,27 ± 6,20	73,07 ± 1,74	85,92 ± 2,06	80,68 ± 8,81	77,71 ± 6,05	78,80 ± 5,88	70,00 ± 3,92	31,42 ± 9,03
**Molarity (mM)**	0,43	0,41	0,28	0,28	0,33	0,31	0,30	0,30	0,27	0,12
**conversion**	86%	82%	56%	56%	66%	62%	60%	60%	54%	24%

Table S5. Yields of biotransformations supplemented with detergents. Indigo yields and conversions at 72 hours in biotransformations with 1.0 mM indigo, cells from early growth phase concentrated at OD_{730} 4, under 100 μE, and growth medium supplemented with different detergents at different percentage (from 0% to 0.5%). Three independent replicated were examined.

Adsorbent	Yield (mg/L)
**Polyamide netted film**	80,5 ± 6,4
**Bio-Beads SM2**	53,7 ± 8,3
**Expanded polyethylene**	48,2 ± 11,3

Table S6. Yields of indigo recovered by different adsorbents.

Supplementary References

Böhmer S., Köninger K., Gómez-Baraibar Á., Bojarra S., Mügge C., Schmidt S., Nowaczyk M.M., and Kourist R. (2017) Enzymatic oxyfunctionalization driven by photosynthetic water-splitting in the cyanobacterium *Synechocystis* sp. PCC 6803. *Catalysts* **7**: 240.

Ng A.H., Berla B.M.,and Pakrasi H.B. (2015) Fine-tuning of photoautotrophic protein production by combining promoters and neutral sites in the cyanobacterium *Synechocystis* sp. strain PCC 6803. *Appl. Environ. Microbiol*. **81**: 6857–6863.

Singh A.K., Elvitigala T., Cameron J.C., Ghosh B.K., Bhattacharyya-Pakrasi M., and Pakrasi H.B. (2010). Integrative analysis of large scale expression profiles reveals core transcriptional response and coordination between multiple cellular processes in a cyanobacterium. *BMC Syst. Biol*. **4**: 105.

Zhou J., Zhang H., Meng H., Zhu Y., Bao G., Zhang Y., Li Y., and Ma Y. (2014) Discovery of a super-strong promoter enables efficient production of heterologous proteins in cyanobacteria. *Sci. Rep.* **4**: 1–6.
